# Complete genome sequence of multidrug-resistant *Edwardsiella ictaluri* A21-00102 isolated from channel catfish (*Ictalurus punctatus*) in the United States

**DOI:** 10.1128/mra.01315-25

**Published:** 2026-06-29

**Authors:** Rideeta Islam Aishy, Fenny Patel, Anna Collinsgru, Mark L. Lawrence, Larry A. Hanson, Stephen R. Reichley, Hasan C. Tekedar

**Affiliations:** 1 Department of Comparative Biomedical Sciences, College of Veterinary Medicine, Mississippi State University 5547, Mississippi State, Mississippi, USA; 2 Global Center for Aquatic Health and Food Security, Mississippi State University 5547, Mississippi State, MS, USA; 3 Department of Pathobiology and Population Medicine, College of Veterinary Medicine, Mississippi State University 5547, Mississippi State, Mississippi, USA; Fluxus Inc., Sunnyvale, California, USA

**Keywords:** *Edwardsiella ictaluri*, multidrug resistance, aquaculture, aquaculture pathogens, catfish

## Abstract

*Edwardsiella ictaluri* is the causative agent of enteric septicemia of catfish, a major disease associated with high mortality and economic loss in U.S. aquaculture. Here, we report the complete genome sequence of *E. ictaluri* A21-00102, a multidrug-resistant strain, consisting of a chromosome of 3,829,734 bp and three plasmids.

## ANNOUNCEMENT

*Edwardsiella ictaluri* is a gram-negative facultative anaerobe and the causative agent of enteric septicemia of channel catfish, a disease associated with high mortality and significant economic losses in commercial catfish aquaculture. It also causes disease in other fish species, including zebrafish, tilapia, and ayu (1–3). Here, we report the complete genome sequence of multidrug-resistant *E. ictaluri* strain A21-00102.

Strain A21-00102 was recovered in 2021 from an aseptically collected diagnostic posterior kidney swab from a diseased, farm-raised channel catfish in Mississippi and submitted to the Mississippi State University Veterinary Microbiology Laboratory. The swab was streaked onto Remel Tryptic Soy Agar with 5% sheep blood (Thermo Scientific; catalog no. R01202) and incubated at 25°C for 48 h. A representative colony was subcultured for purity. Oxidase and indole testing followed by Sensititre ARIS 2X biochemical identification confirmed *E. ictaluri* with 97.62% probability.

Broth microdilution susceptibility testing was performed in 96-well plates using twofold antibiotic dilutions in Luria-Bertani, inoculated with mid-log-phase *E. ictaluri* A21-00102, and incubated at 28°C for 24 h; MICs were defined as ≥90% growth inhibition. Strain A21-00102 showed MICs of 22.24 µg/mL (florfenicol), 15.9 µg/mL (oxytetracycline), and 18.26 µg/mL (Romet). For reference strain *E. ictaluri* 93-146, the Romet MIC was 9.13 µg/mL; florfenicol and oxytetracycline MICs were not determined because the strain showed insufficient growth despite repeated testing at very low antibiotic concentrations (4, 5).

For DNA extraction, *E. ictaluri* A21-00102 was cultured in LB broth at 28°C for 24 h. Cells were harvested, washed once with phosphate-buffered saline (PBS), and submitted to Plasmidsaurus in 500 µL Zymo 1× DNA/RNA Shield. DNA was extracted using the ZymoBIOMICS 96 MagBead DNA Kit. ONT libraries were prepared using the Rapid Barcoding Kit 96 V14 (SQK-RBK114.96) and sequenced on a PromethION P24 with an R10.4.1 flow cell. Basecalling used ont-dorado-for-promethion in super-accurate mode with a minimum *Q* score of 10, and adapters were trimmed using MinKNOW. Illumina libraries were prepared using the Illumina DNA Prep Kit (part no. 20060059) and sequenced on a NovaSeq X Plus to generate paired-end 2 × 150 bp reads. A total of 8,495,928 raw Illumina reads were generated, and 8,283,604 remained after processing with fastp v0.24.0 (6).

ONT reads were assembled using Autocycler (7) with Flye v2.9.6+ (8), Hifiasm (9), and Plassembler v1.8.0+ (10). The circular chromosome was rotated using dnaapler so that base 1 corresponded to the start of the dnaA gene (11), polished with Medaka v1.8.0 (12), and hybrid-polished with Illumina reads using bwa-mem2 (13) and Polypolish v0.6.0 (14). Default parameters were used unless otherwise stated. NCBI PGAP v6.10 was used for annotation (15). Assembly structure was visualized with Bandage v0.8.1 (16), genome quality was assessed using CheckM v1.2.2 (17), and species/plasmid identification was performed using Mash v2.3 and Sourmash v4.6.1 (18, 19). The final assembly consisted of one circular chromosome and three circular plasmids; features are listed in Table 1.

**TABLE 1:** Genome assembly and annotation statistics for *Edwardsiella ictaluri* A21-00102^*a*^

Feature	Value
Total genome size	3,968,950 bp
Chromosome	3,829,734 bp; circular
Plasmid 1	118,316 bp; circular
Plasmid 2	11,286 bp; circular
Plasmid 3	9,614 bp; circular
GC content	57.10%
Total predicted genes	3,778
Coding sequences	3,651
Protein-coding genes	3,420
RNA genes	127
rRNAs	25 total: 9 5S, 8 16S, and 8 23S
tRNAs	97
ncRNAs	5
Pseudogenes	231
ONT reads	180,718
ONT bases	891,129,824 bp
ONT read N50	6,989 bp
Longest ONT read	60,029 bp
ONT raw coverage	~224×
Final assembled coverage	~97×
Raw Illumina reads	8,495,928
Illumina reads after fastp QC	8,283,604

^*a*^ ONT, Oxford Nanopore Technologies; QC, quality control; rN50, read N50; rRNA, ribosomal RNA; tRNA, transfer RNA; ncRNA, noncoding RNA; and GC, guanine-cytosine.

CARD identified sul2, floR, aph(3ʺ)-Ib, and aph (6)-Id on the 118-kb plasmid (20). These data provide a genome resource for multidrug-resistant *E. ictaluri* from U.S. farm-raised channel catfish.

## Data Availability

This Whole Genome Shotgun project is deposited in DDBJ/ENA/GenBank under JBQERV000000000. The chromosome accession is CM125427.1, and the three plasmid accession numbers are JBQERV010000003.1, JBQERV010000004.1, and JBQERV010000002.1. The genome is associated with BioProject PRJNA1302501 and BioSample SAMN50480338. Raw sequencing data for *Edwardsiella ictaluri* A21-00102 are publicly available in the NCBI Sequence Read Archive (SRA) under accession numbers SRR36076355 for Illumina sequencing data and SRR36076354 for Nanopore sequencing data.

## References

[1] Soto E, Griffin M, Arauz M, Riofrio A, Martinez A, Cabrejos ME. 2012. *Edwardsiella ictaluri* as the causative agent of mortality in cultured *Nile tilapia*. J Aquat Anim Health 24:81–90. doi:10.1080/08997659.2012.675931 22838078

[2] Nagai T, Iwamoto E, Sakai T, Arima T, Tensha K, Iida Y, Iida T, Nakai T. 2008. Characterization of *Edwardsiella ictaluri* isolated from wild ayu *Plecoglossus altivelis* in Japan. Fish Pathol 43:158–163. doi:10.3147/jsfp.43.158

[3] Hawke JP, Kent M, Rogge M, Baumgartner W, Wiles J, Shelley J, Savolainen LC, Wagner R, Murray K, Peterson TS. 2013. Edwardsiellosis caused by *Edwardsiella ictaluri* in laboratory populations of zebrafish *Danio rerio*. J Aquat Anim Health 25:171–183. doi:10.1080/08997659.2013.782226 23865817 PMC4077847

[4] Gaunt PS, McGinnis AL, Santucci TD, Cao J, Waeger P, Endris RG. 2006. Field efficacy of florfenicol for control of mortality in channel catfish, *Ictalurus punctatus* (Rafinesque), caused by infection with *Edwardsiella ictaluri*. J World Aquaculture Soc 37:1–11. doi:10.1111/j.1749-7345.2006.00001.x

[5] Gaunt PS, Gao D, Hawke JP, Griffin MJ, Ware C, Endris RG. 2021. Minimal inhibitory concentration values of oxytetracycline for bacterial pathogens isolated from warmwater fishes. N Am J Aquac 83:138–144. doi:10.1002/naaq.10166

[6] Chen S, Zhou Y, Chen Y, Gu J. 2018. Fastp: an ultra-fast all-in-one FASTQ preprocessor. Bioinformatics 34:i884–i890. doi:10.1093/bioinformatics/bty560 30423086 PMC6129281

[7] Wick RR, Howden BP, Stinear TP. 2025. Autocycler: long-read consensus assembly for bacterial genomes. Bioinformatics 41:btaf474. doi:10.1093/bioinformatics/btaf474 40875535 PMC12460055

[8] Kolmogorov M, Yuan J, Lin Y, Pevzner PA. 2019. Assembly of long, error-prone reads using repeat graphs. Nat Biotechnol 37:540–546. doi:10.1038/s41587-019-0072-8 30936562

[9] Cheng H, Concepcion GT, Feng X, Zhang H, Li H. 2021. Haplotype-resolved *de novo* assembly using phased assembly graphs with hifiasm. Nat Methods 18:170–175. doi:10.1038/s41592-020-01056-5 33526886 PMC7961889

[10] Bouras G, Sheppard AE, Mallawaarachchi V, Vreugde S. 2023. Plassembler: an automated bacterial plasmid assembly tool. Bioinformatics 39:btad409. doi:10.1093/bioinformatics/btad409 37369026 PMC10326302

[11] Bouras G, Grigson SR, Papudeshi B, Mallawaarachchi V, Roach MJ. 2024. Dnaapler: a tool to reorient circular microbial genomes. JOSS 9:5968. doi:10.21105/joss.05968

[12] Technologies ON. 2018. Medaka v1.8.0: sequence correction provided by oxford nanopore technologies, v1.8.0. GitHub. https://github.com/nanoporetech/medaka.

[13] Vasimuddin M, Misra S, Li H, Aluru S. 2019. Efficient architecture-aware acceleration of BWA-MEM for multicore systems. 2019 IEEE International Parallel and Distributed Processing Symposium (IPDPS); Rio de Janeiro, Brazil. IEEE Computer Society. doi:10.1109/IPDPS.2019.00041

[14] Wick RR, Holt KE. 2022. Polypolish: short-read polishing of long-read bacterial genome assemblies. PLoS Comput Biol 18:e1009802. doi:10.1371/journal.pcbi.1009802 35073327 PMC8812927

[15] Tatusova T, DiCuccio M, Badretdin A, Chetvernin V, Nawrocki EP, Zaslavsky L, Lomsadze A, Pruitt KD, Borodovsky M, Ostell J. 2016. NCBI prokaryotic genome annotation pipeline. Nucleic Acids Res 44:6614–6624. doi:10.1093/nar/gkw569 27342282 PMC5001611

[16] Wick RR, Schultz MB, Zobel J, Holt KE. 2015. Bandage: interactive visualization of de novo genome assemblies. Bioinformatics 31:3350–3352. doi:10.1093/bioinformatics/btv383 26099265 PMC4595904

[17] Parks DH, Imelfort M, Skennerton CT, Hugenholtz P, Tyson GW. 2015. CheckM: assessing the quality of microbial genomes recovered from isolates, single cells, and metagenomes. Genome Res 25:1043–1055. doi:10.1101/gr.186072.114 25977477 PMC4484387

[18] Ondov BD, Treangen TJ, Melsted P, Mallonee AB, Bergman NH, Koren S, Phillippy AM. 2016. Mash: fast genome and metagenome distance estimation using MinHash. Genome Biol 17:132. doi:10.1186/s13059-016-0997-x 27323842 PMC4915045

[19] Pierce NT, Irber L, Reiter T, Brooks P, Brown CT. 2019. Large-scale sequence comparisons with sourmash. F1000Res 8:1006. doi:10.12688/f1000research.19675.1 31508216 PMC6720031

[20] Alcock BP, Raphenya AR, Lau TTY, Tsang KK, Bouchard M, Edalatmand A, Huynh W, Nguyen A-LV, Cheng AA, Liu S, et al.. 2020. CARD 2020: antibiotic resistome surveillance with the comprehensive antibiotic resistance database. Nucleic Acids Res 48:D517–D525. doi:10.1093/nar/gkz935 31665441 PMC7145624

